# Seasonal Morphological and Biochemical Variation of *Coffea canephora* Pierre ex A. Froehner (Rubiaceae) Leaves of Early, Intermediate and Late Maturing Genotypes

**DOI:** 10.3390/plants13243461

**Published:** 2024-12-11

**Authors:** Jeane Crasque, Jean Marcel Sousa Lira, Giuseppe Tognere Polonini, Thiago Corrêa de Souza, Edilson Romais Schmildt, Lúcio de Oliveira Arantes, Sara Dousseau-Arantes

**Affiliations:** 1 Instituto Capixaba de Pesquisa, Assistência Técnica e Extensão Rural—Centro Regional de Desenvolvimento Rural—Norte, Linhares 29901-443, ES, Brazil; tognerepoloninig@gmail.com (G.T.P.); lucio.arantes@incaper.es.gov.br (L.d.O.A.); 2 Setor de Fisiologia Vegetal, Departamento de Biologia, Universidade Federal de Lavras, Lavras 37200-900, MG, Brazil; jmslira1283@gmail.com; 3 Instituto de Ciências da Natureza—ICN, Universidade Federal de Alfenas, UNIFAL, Alfenas 37130-001, MG, Brazil; thiagonepre@hotmail.com; 4 Departamento de Ciências Agrárias e Biológicas, Universidade Federal do Espírito Santo, CUNES, São Mateus 29932-540, ES, Brazil; e.romais.s@gmail.com

**Keywords:** climate, coffee, critical points, logistic model, photoperiod

## Abstract

Understanding the growth patterns of genotypes optimizes their selection and management. The objective of this study is to investigate the seasonal variations in the morphology and biochemistry of *Coffea canephora* clone leaves, considering climatic conditions and the maturation cycle. Morphological characteristics and carbohydrate contents of the leaves were analyzed throughout the growth cycle. A nonlinear logistic model was applied, and critical points of the leaf emission rates of plagiotropic branches were determined. Leaf growth was greater at higher temperatures during the rainy periods and lower at milder temperatures during the dry season. Genotype 143 exhibited the largest leaf width in spring, while 104, A1, and P2 had the largest leaf width in summer. The logistic model was suitable for describing leaf emission, with the critical points of genotype 143 being earlier, while P2 displayed a longer leaf emission cycle. The peak growth period influenced the quantities of starch and total soluble sugars in the leaves. The dormancy period showed a higher availability of reducing sugars. Pearson correlation indicated significant coefficients between temperature, precipitation, photoperiod, and foliar characteristics. The results obtained serve as a reference for future investigations, particularly in response to environmental challenges.

## 1. Introduction

Coffee is the second most traded commodity in the world, just behind petroleum products, with *Coffea arabica* and *Coffea canephora* accounting for 99% of global production. Brazil stands out as the largest coffee producer [1], but in the Brazilian regions that cultivate *C. canephora*, there is already a reduction in rainfall and an increase in dry periods. Additionally, there has been an increase in the frequency of heat waves, resulting in significantly high temperatures [2]. In this context, the leaves, which are essential for photosynthesis and plant health, will be severely affected by these changes, leading to a reduction in coffee productivity [3].

The leaf captures sunlight and synthesizes photo-assimilates, consequently contributing to productivity [4]. In Brazil, most of the *C. canephora* crops are grown in full sun, where high temperatures, radiation intensity, and water deficit are frequent throughout the seasons. However, leaves of *C. canephora* may be tolerant to heat [5,6], but high intensity associated with the reduction in water availability, caused by climate change, may have significant consequences on productivity and quality [2,7,8].

*Coffea canephora* exhibits high genetic variability in its vegetative and reproductive development [9], with fruits that display distinct growth patterns and maturation times [10]. Although knowledge about clonal varieties of different maturation cycles is on the rise, many assessments do not consider this characteristic. Furthermore, simultaneous vegetative development during fruiting can lead to bienniality in production, as the imbalance in fruit load can compromise subsequent flowering [11].

Coffee leaf patterns vary seasonally in response to climatic conditions, with higher growth rates during rainy periods and high temperatures, and lower rates during drought [12]. Low water availability is the main signal of leaf abscission [13]. In addition, under high light, coffee trees increase the number of leaves, leaf area, and plant biomass. In contrast, in short photoperiods and low solar radiation, there is a decrease in the size of the leaves and an increase in thickness, specific dry mass, and starch content [8,14,15]. This leaf dynamics is very important for growth and, consequently, fruit production.

Identifying leaf patterns is important for a better understanding of crops in a climate change scenario. Analyses with nonlinear models have been conducted to investigate the accumulation patterns of dry matter, macronutrients, and micronutrients of fruits and leaves in *C. canephora* [16,17,18,19,20]. The Logistic and Gompertz models are the most used, and the Logistic model is preferentially used due to its better fit [21]. Through these models, it is possible to determine the highest growth rates and critical points, such as inflection points (IP), maximum acceleration (MAP), minimum deceleration (MDP), and asymptotic deceleration (ADP). To date, no studies have been conducted describing the critical points and the seasonal leaf pattern in different genotypes of *C. canephora*.

Seasonal variations in the morphology and biochemistry of coffee leaves are controlled by climatic conditions, availability of water resources, and the stage of maturation of the plant. However, the comprehensive understanding of the specific effects of these factors on early, intermediate, and late maturation genotypes of *C. canephora* is still limited. Knowledge of the process of leaf emission and expansion and its seasonal variations is important to establish management techniques to improve productivity. The complex interaction between climatic factors in the formation of leaf area and in the distribution of carbohydrates throughout the year directly interferes with production. The hypothesis is that there are distinct seasonal patterns in leaf morphology and biochemistry among *Coffea canephora* genotypes, influenced by climatic conditions and the maturation cycle. The objective of this study is to investigate the seasonal variations in the morphology and biochemistry of *Coffea canephora* clone leaves, considering climatic conditions and the maturation cycle.

## 2. Materials and Methods

### 2.1. Study Location

The experiment was carried out at the Experimental Farm of the Capixaba Institute of Research, Technical Assistance and Rural Extension (INCAPER), located in the municipality of Marilândia, Espírito Santo, Brazil (latitude of 19°24′19″ S, longitude of 40°32′20″ W and 188 m of altitude). The soil is classified as dystrophic Oxisol [22], and the climate is tropical, categorized as Aw according to the Köppen and Geiger classification [23].

### 2.2. Description of Culture

A 3-year-old crop was used, cultivated in full sun with a spacing of 3 m × 1.5 m, in the rainfed system, without a supplemental irrigation system. Genotypes of different early (104 and A1), intermediate (P2), and late (143) maturation cycles were evaluated.

### 2.3. Climatic Conditions

The weather conditions were obtained by the automatic weather station, belonging to the National Institute of Meteorology (INMET). The daily data for the entire evaluation period of the photoperiod was obtained from the Solar Topo electronic platform [24], and the photoperiod calculation was conducted for the specified month. Potential evapotranspiration (Etp) was obtained by the [25] method. The meteorological variables analyzed were between August 2021 and August 2022, and include average temperature (°C), minimum temperature (°C), maximum temperature (°C), average relative humidity (%), solar radiation (KJ/m^2^), accumulated precipitation (mm), potential evapotranspiration (mm $d^{-1}$) and photoperiod.

### 2.4. Vegetative Growth Assessments

After the 2021 harvest, two primary plagiotropic branches, emerging from the orthotropic branches in the upper third of the plant canopy, were selected to evaluate the number of leaves of the plagiotropic branches (NF) by direct count. In addition, the seasonal growth rate was determined by the initial and final value of each season.

The evaluations of leaf expansion of the limbus were carried out at all solstices and equinoxes of the year with the aid of a graduated ruler, in 12 plants of each genotype. The evaluations began on the following dates: 11 August 2021, 4 October 2021, 1 February 2022, and 26 April 2022. Two young leaves in the expansion phase were selected in the upper part of the canopy of each plant. The calculations were performed as described by [26]:

(1) LA=0.667×LL×LW,

where LA corresponds to estimated leaf area (cm^2^); LL is the length of the central rib of the sheet (cm) and LW is the maximum width of the leaf blade (cm). With the results of the estimated leaf area, the rate of expansion of the daily leaf area was determined (DLAFE, ${\mathrm{cm}}^{2}$ $d^{-1}$) in every season of the year.

(2) DLAFE=(lnAf2−lnAf1)/t2−t1

Af1 and Af2 represent the leaf areas (cm^2^) measured at two distinct times t1 and t2; t1 and t2 are the corresponding times of the evaluations, allowing for the calculation of the growth rate of leaf area over the time interval. At the end of each evaluation of the leaf area of the leaves in the season, the leaves were collected and washed to be dried in an oven at 65 °C until they reached constant weight, then the leaf dry mass (LDM) was obtained on an analytical scale and the specific leaf area (SLA) was obtained by the leaf area/mass ratio.

### 2.5. Carbohydrate Allocation

Carbohydrate allocation was evaluated by quantifying reducing sugars (RS), total soluble sugars (TSS), and leaf starch of each season, at harvest and after harvest Table 1.

The dried plant materials were ground in a Willeve knife mill model STAR FT-50, and stored in a freezer at −18 °C. The extracts were obtained according to [27], using 0.05 g for the late 55 after harvest (20/set (T55)) and 0.07 g for the other seasons. Starch extraction was carried out using the method of [12] with modifications. 0.5 mL (60,000 U/µmL) of thermostable a-amylase (A3306, Sigma-Aldrich Inc., St. Louis, MO, USA) diluted in buffer in potassium phosphate buffer 100 mM pH 6.5 were added. The samples were incubated at 75 °C for 30 min. This procedure was repeated once again totaling 60,000 enzyme units. The samples were cooled to 50 °C, and then 0.5 mL of a solution containing 260 U/µmL of amyloglucosidase (A7095, Sigma-Aldrich Inc.) was added to 200 mM potassium acetate buffer pH 4.5. The samples were incubated at 50 °C for 30 min. This procedure was repeated once again. After the incubations described above, 100 µmL of 0.8 M perchloric acid was added to stop the reaction and precipitate proteins. For the quantification of TSS and starch, the Antrona method was used [28], with modifications, using 2 mL of the 0.19% antrona solution in 93.33% sulfuric acid, in a reaction volume of 3 mL, subjected to 100 °C for 3 min. The RS were quantified according to the protocol described by [29], using the Dinitrosalicylic Acid (DNS) method.

### 2.6. Univariate Logistic Model and Critical Point Analysis in Growth Dynamics

For the univariate Logistic model, the following expression was used: yi = *a*/[1 + exp(−*b* − *c*x)], where yi represents the ith observation of the dependent variable, where i = 1, 2, …, n; *a* is the asymptotic value or final growth value; *b* is the allocation parameter of the curve; *c* is the maximum relative growth rate or index of precocity; and x is the independent variable. The critical points of the model that was selected as the best in each treatment were calculated, namely the maximum acceleration point (MAP), the inflection point (IP), the maximum deceleration point (MDP), and the asymptotic deceleration point (ADP) for all genotypes according to the methodology of [30,31], as presented in Table 2 with the following equations:

The normality of the residuals was verified using the Shapiro–Wilk test. The null hypothesis of this test postulates that the residues follow a normal distribution. In addition, the homogeneity of the variance of the residuals was evaluated using the Breusch–Pagan test. The null hypothesis in this case is that the residuals are homoscedasticity, that is, that the variance of the residuals is constant in relation to the independent variables. To verify the presence of first-order residual autocorrelation, the Durbin–Watson test was used, whose null hypothesis presupposes independence between the residuals.

To evaluate the goodness of fit of the models, the following indicators were calculated: coefficient of determination ($R^{2}$), adjusted coefficient of determination ($R_{aj}^{2}$), Akaike information criterion (AIC). The best-fit models are those with higher values of $R^{2}$ and $R_{aj}^{2}$, closer to 1, and lower values of AIC.

### 2.7. Experimental Design and Statistical Analysis

The adopted design was completely randomized and used three replicates of four plants in a split-plot scheme, where the plot was represented by the genotypes of different maturation cycles (early, intermediate, and late) and the subplot was the evaluation times. The data were submitted for analysis of variance and the means were compared by the Scott–Knott test at 5% probability. The relationship between the meteorological data, considered explanatory characteristics, and response variables analyzed, was obtained through principal component analysis (PCA) and Pearson’s correlation, considering the winter, spring, summer, and autumn seasons. Seasonal analysis is significant because it provides a comprehensive understanding of how genotype responses vary under different climatic conditions, offering insights into genotypes’ response to climate variability. Correlations with monthly climate variables were calculated from August to June (2021–2022). The analyses were performed using the R software version 4.2.1 through the packages and ExpDes.pt for statistical analyses and Pearson’s correlation analyses were performed using the PAST statistical program [2].

## 3. Results

Table 3 highlights the climatic conditions, showing an average annual air temperature of 24.26 °C, with monthly averages ranging from a minimum of 15.28 °C in June 2022. The coldest months occurred between April and August, while the months of January and February recorded the highest temperatures (33.5 °C), solar radiation (above 1500 KJ/m^2^), and potential evapotranspiration (above 160 mm $d^{-1}$). January also had the longest length of the day. In September, there was a peak in humidity, reaching 65.10%. Annual rainfall totaled 972.80 mm, with rainfall between October and February accounting for 86% of the annual total.

All assumptions were met with a *p*-value higher than 0.05 Table 4. For the Logistic model, the coefficients of determination ($R^{2}$) ranged from 0.97 to 0.99, and the adjusted coefficient of determination ($R_{aj}^{2}$) was above 0.96. They are considered adequate, as they have higher values of $R^{2}$ and $R_{aj}^{2}$, closer to 1 [31], and lower values of AIC.

**Figure 1:**
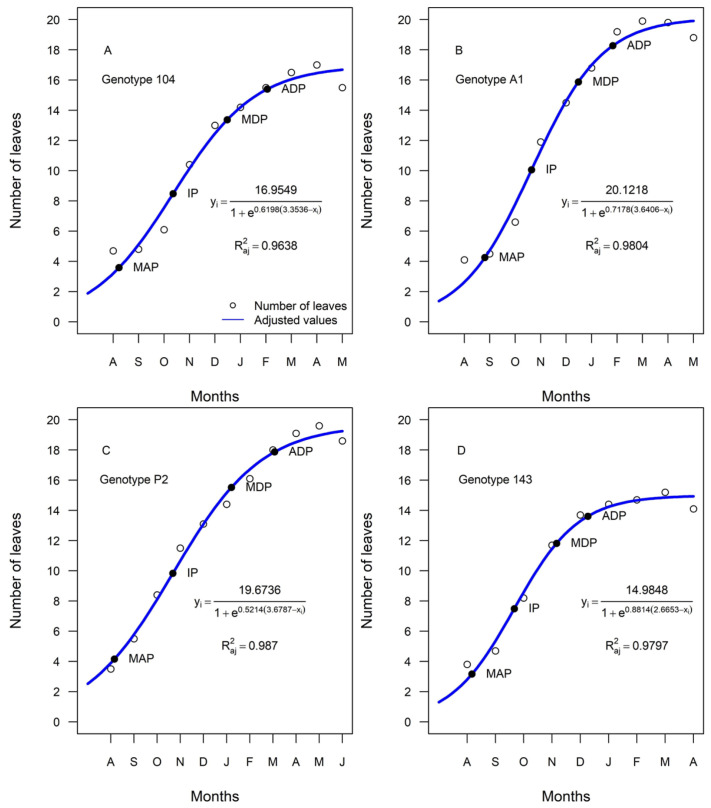
Growth curves adjusted by the Logistic model for the number of leaves of the plagiotropic branches of the early maturation genotypes 104 and A1, intermediate P2, and late 143. (**A**). Genotype 104. (**B**). Genotype A1. (**C**). Genotype P2. (**D**). Genotype 143.

The curve data were analyzed considering the harvest period of each genotype and the beginning of defoliation. The maximum acceleration point (MAP) was identified in August (Figure 1). The tipping point (IP) varies between genotypes, with early and intermediate genotypes occurring in October and late in September. At the maximum deceleration point (MDP), the early genotypes reached in December, the intermediate genotypes in February, and the late genotype in November. The identification of the asymptotic deceleration point (ADP) occurred in February for the early genotypes, in March for the intermediate genotypes, and in December for the late genotypes. Regarding the beginning of the defoliation period, the early genotypes started in May, the intermediate ones in June, and the late genotype in April.

### 3.1. Leaf Evaluation

For all variables, no significant difference was observed between the genotypes in the seasons, with the exception of the variable maximum width of the leaf blade (Table 5). In winter, there was no difference between the genotypes for maximum width of the leaf blade (mean 1.28 cm), while in spring, genotype 143 showed the maximum width of the leaf blade (3.03 cm), followed by genotype P2 (2.35 cm), and early genotypes 104 and A1 showed no differences between them (mean of 1.53 cm). In the summer, the P2 genotype obtained the highest maximum width of the leaf blade (3.11 cm), while the others did not show differences (mean of 2.29 cm). In autumn, the highest averages were observed for genotypes A1 and P2 (mean 2.26 cm), followed by genotypes 104 and 143 (mean 1.58 cm).

When evaluating within the genotype level, for the early genotypes, genotype 104 presented the highest maximum width of the leaf blade in the summer, and the A1 genotype in the summer and autumn, with no statistical differences between the other seasons. The P2 genotype had a higher maximum width of the leaf blade in the summer and lower in the winter. Genotype 143 showed the highest maximum width of the leaf blade in spring, followed by summer, with no statistical differences between autumn and winter.

Evaluating the seasons during the summer period, an increase in the values of the variables leaf central vein length, leaf area, and daily leaf area expansion rate was observed, while these decreased during the winter (Table 5). No statistically significant differences were observed between the autumn and spring seasons for these variables. Specific leaf area was higher in winter, followed by summer, while spring and autumn did not show statistically significant differences. As for leaves of plagiotropic branches, the lowest value was observed in spring, followed by summer, autumn, and winter. The leaf dry mass was lower in the winter.

Within the genotype level, no statistically significant differences were identified between the genotypes at the stations in relation to the leaf central vein length and leaves of plagiotropic branches variables (Table 5).

The P2 genotype stood out with the highest values of leaf area, daily leaf area expansion rate, and leaf dry mass, while the other genotypes did not show significant differences between them. Regarding the specific leaf area variable, genotypes A1 and 143 presented the highest values observed.

### 3.2. Carbohydrates

The genotypes did not show significant differences between them during the evaluation periods in the starch and TSS variables (Table 6). The highest TSS content for all genotypes was observed during spring and autumn, followed by the other seasons, which did not show significant differences between them. Starch had the highest values in the summer, followed by the autumn. As for RS, the A1 genotype stands out in winter and spring with the highest averages. At harvest, genotype 143 presented the lowest averages. There are no significant differences between the genotypes in summer and fall. Evaluating the genotypes within each season, the winter had the highest RS values, and at harvest, genotypes P2 and 143 also presented the highest averages (Table 6).

During the evaluation times after harvest, T55 had higher values of starch and TSS, and P2 had higher TSS. The lowest values were observed in the summer, at harvest, and in P55 (Table 7). In relation to RS, the A1 genotype showed the highest mean in T55. The P2 and 143 genotypes did not vary significantly between P55, I55, and T55. Genotype 143 had the highest averages in I55 and T55.

### 3.3. Pearson Correlation Coefficients and Principal Components Analysis (PCA)

The climatic variables relate to the variables leaf central vein length, leaf area, and daily leaf area expansion rate, presenting positive and statistically significant correlations with all the climatic parameters considered, with the exception of the mean temperature (Table 8). On the other hand, specific leaf areas demonstrated a negative correlation with photoperiod and humidity. Leaves of plagiotropic branches, in turn, showed a positive correlation with minimum temperature, humidity, and precipitation. As for the maximum width of the leaf blade variable, a positive correlation was observed with most climatic parameters, except for maximum and average temperature. With regard to carbohydrates, a significantly positive correlation was found between ETp, radiation, and maximum temperature with starch content, while TSS showed a negative correlation with maximum temperature. RS obtained a significant negative correlation with most climatic variables except for maximum and mean temperature.

**Figure 2:**
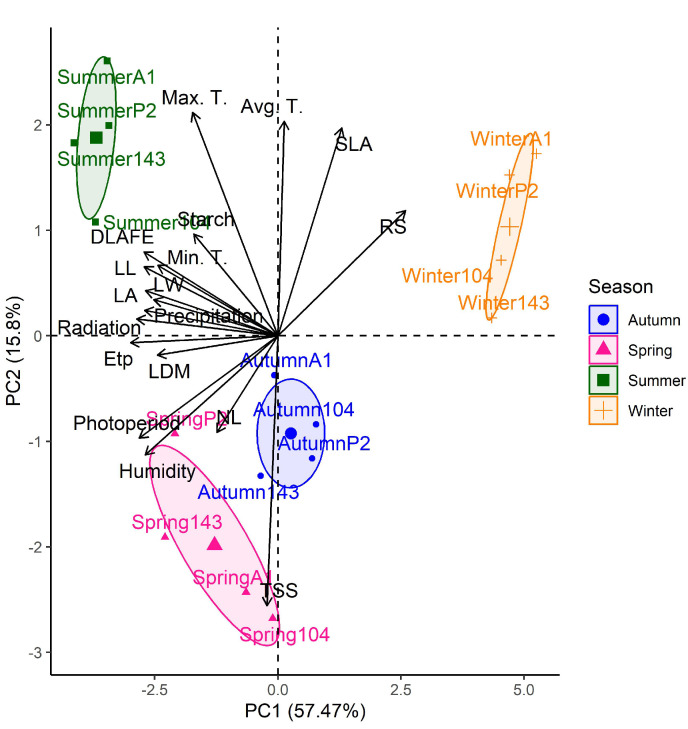
Principal component analysis (PCA) of the mean values of leaf variables and climatic factors: maximum temperature (Max. T.); minimum temperature (Min. T.); average temperature (Avg. T.); average relative humidity (Humidity); accumulated precipitation (Precipitation); solar radiation (Radiation); potential evapotranspiration (ETp); photoperiod (Photoperiod); leaf central vein length (LL); maximum leaf blade width (LW); leaf area (LA); daily leaf area expansion rate (DLAFE); leaf dry mass (LDM); specific leaf area (SLA); leaves of plagiotropic branches (NL); reducing sugars (RS); total soluble sugars (TSS) and starch of early maturation genotypes 104 and A1, intermediate P2 and late 143 in four seasons of the year.

A Principal Component Analysis (PCA) was conducted using data with all genotypes throughout the seasons. The PCA results revealed that 73% of the total variability in the data was explained by Principal Components 1 (PC1) and 2 (PC2) (Figure 2). The environmental and leaf variables showed a seasonal distribution, grouped into winter-autumn and spring-summer periods, with the growth variables showing greater proximity to precipitation, radiation, humidity, minimum temperature, and photoperiod during spring and summer. Carbohydrate contents were associated with winter for RS, spring for TSS, and summer for starch.

## 4. Discussion

### 4.1. Univariate Logistic Model and Critical Point Analysis in Growth Dynamics

The estimated critical points represent valuable tools in the management of the conilon coffee crop. The maximum acceleration point (MAP) marks the beginning of plant growth [32], characterized by a significant increase in the number of leaves in this study, being observed in August. The A1 genotype has a later onset of growth, occurring at the end of August, (at 8 weeks), compared to the other genotypes. This indicator is crucial as it indicates before the MAP that plants have a reduced growth capacity [32].

The difference observed at the beginning of the defoliation period between the early, intermediate, and late genotypes may have influenced the occurrence of an earlier inflection point (IP) for genotype 143 and consequently the defoliation period. The PI is the moment when the growth rate reaches its maximum, characterized by the emission of a greater amount of leaves by the plants [32].

Considering the early onset of PI and defoliation of genotype 143, it is possible to infer that this genotype has different growth dynamics in relation to the other genotypes. In addition, genotype 143 obtained fewer final leaves than the other genotypes. In this sense, smaller branches, fewer leaves, and/or smaller leaves have fewer photoassimilates available to sustain growth [33]. Reference [34] observed that the decline in tree growth induced by defoliation is due to limitations in carbon production and consumption, with a more pronounced impact in cases of intense defoliation.

The anticipation of PI in the late genotype can influence agronomic management, such as fertilization, requiring better programming to optimize practices and maximize the productivity of conilon coffee. If any intervention in crop growth is needed, such as fertilization, it will be more effective between MAP and IP [35]. According to [36], nitrogen and potassium are the nutrients that most limit coffee production. At the beginning of growth, these nutrients have a greater absorption, resulting in a rapid increase in nutritional demand, a period that can be compared to the interval between the maximum acceleration points (MAP) and inflection points (IP).

The maximum deceleration point (MDP) corresponds to the point at which the growth rate of the function reaches its maximum [32], indicating the moment when the growth of the curve slows to the maximum before stabilizing. During the period between the IP point and the MDP, there was an increase in precipitation (from October to February) and a long photoperiod (above 12 h). Reference [14] shows that the photoperiod of short days affects plant growth by reducing the number of leaves, thickness, chlorophyll content, and dry matter. However, studies indicate that the presence of fruits on the branches affects more than the change in the photoperiod [12], but regardless of the long photoperiod regime or not between mid-March and early May, it coincides with high drops in the growth of branches and leaf area.

Asymptotic deceleration point (ADP), is the point at which the growth rate of the function stabilizes and approaches zero. After ADP, excess nutrition will not significantly promote crop growth [35]. The identification of ADP becomes important because it allows predicting the end of the crop cycle.

The phenology of defoliation in coffee is poorly documented for coffee, and the beginning of defoliation occurred in April for late and May for early. However, it is notable that the P2 genotype differs from this pattern, demonstrating a later onset of defoliation, specifically in June. In addition, the P2 genotype exhibits a higher leaf area, expansion rate, and dry mass compared to other genotypes evaluated. Tolerant genotypes can extend the duration of new leaves, maintaining a larger leaf and root area throughout the growth cycle [37,38]. Reference [37] studied the diversity of root characteristics and observed that the P2 genotype had the highest root volume and A1 the highest amount of deep root. These observations, combined with the larger leaf area associated with the P2 genotype, highlight its relevance for genetic improvement studies aimed at improving the performance of cultivars. Genotypes such as P2 and A1 are often objects of study due to their production potential and vegetative vigor, being considered superior genotypes [37,38,39].

### 4.2. Leaf Evaluation and Carbohydrates

The higher leaf dry mass of the P2 genotype may be related to both its larger leaf area and its higher expansion rate. These combined factors have the potential to significantly increase the plant’s total biomass production, an important aspect of grain production and resilience to environmental stresses. This relationship between leaf area and grain yield was corroborated by [40], who observed that for every 100 cm^2^ of leaf area, there is an estimated increase of 2.37 g of green cherry coffee. These findings highlight the importance of leaf area in coffee productivity and reinforce the relevance of the P2 genotype in terms of yield potential.

Reference [41] highlights the importance of selecting genotypes with larger leaves of *C. canephora* in breeding programs, as this may indicate a yield potential of the crop. Genotypes with larger leaf areas have larger surfaces for light interception, which can result in higher photosynthetic rates and carbohydrate availability [42]. In addition, heat tolerance was positively associated with leaf size [6].

Each genotype can exhibit a distinct growth dynamic. Genotype 143 had its highest growth rate during the spring, while genotypes 104, P2, and A1 showed a significant increase during the summer. This variation suggests that each genotype responds individually to seasonal changes. The beginning and end of the season occur when a limiting factor, such as air temperature, radiation intensity, and water availability, exceeds the threshold below which photosynthesis is restricted [43]. In this sense, the minimum temperatures of winter (15.3 to 16.9 °C) prove to be a limiting factor for leaf development, as it had a low correlation and association with the growth variables. For coffee *C. canephora*, temperatures below 17 °C the growth of the branches is reduced [44] in addition, the rate of photosynthesis decreases, which can lead to productivity drops [45].

In correlation and PCA analysis, it is shown that the leaves develop better during the summer when temperatures are higher and starch is higher. However, the emission of new leaves seems to have a positive relationship with moderate maximum temperatures between 29 and 30 °C. Although *C. canephora* leaves can tolerate a temperature of up to 37 °C by maintaining photoprotection and antioxidant mechanisms [5], extreme temperatures affect productivity by reducing grain weight and yield [45] due to heat stress.

Radiation at the end of the growing season limits growth [43]. Net carbon absorption decreases according to the decrease in radiation intensity during autumn and limits photosynthesis [46], causing the end of the growing season [46], indicates that radiation is the main abiotic limitation in the photosynthetic period that explains the difference between spring and winter.

During the winter, when the plants enter a state of dormancy or reduced growth, a decrease in the leaf area of the coffee plant is observed [47], due to unfavorable environmental conditions for growth, such as low temperatures and lower availability of sunlight. For example, in the present study, it was found that solar radiation during the winter (July and August) is about 33% lower than in the summer (January and February), in addition to a reduced photoperiod.

During spring, they begin a new growth cycle, driven by the increase in temperatures, photoperiod, and radiation observed in the present study. In this study, water availability in spring was also decisive in achieving a higher plagiotropic branch leaves rate and, in summer, a higher leaf area. During this period, it is possible that resources have been directed mainly to the development of new leaves, to the detriment of the greater availability of TSS.

The higher leaf area tends to be associated with a higher seasonal availability of water. These findings are in line with the results presented by [48], who observed higher leaf area values in plants under irrigation. In this sense, it was observed that rainfall was close to the average recorded in the state of Espírito Santo, around 1000 mm [2]), but below the average expected for the months of January to March (137 mm). In these months, the reduction in the volume of rainfall is more harmful than a dry period during the winter [2]. During this period, there is usually a combined effect of drought, heat, and high irradiance, affecting the physiological performance of the crop, especially production [2]. Leaf fall observed in the warmer period may be a common response to water stress in *C. canephora* genotypes, where plants subjected to a water deficit affect the photosynthetic process [13].

In our study, we observed that specific leaf area was the most affected trait, mainly due to variations in light hours. Reductions in the availability of solar luminosity resulted in an increase in specific leaf area. According to the results of [15] compared to coffee plants grown in the shade, those grown in the sun had higher specific leaf area, even with smaller leaf area and total dry mass.

In general, carbohydrate levels increase at the end of winter. During this season, plants tend to direct their resources toward starch storage, as they are in a period of dormancy. Similar results were observed by [49] in *C. arabica* L.

With the increase in temperatures and the prolongation of the days, the plants intensify their metabolic activity, promoting an active growth of leaves and the development of new tissues, which demands a considerable amount of energy derived from soluble sugars [49]. This process is observed by the increase in TSS, especially because in this period it begins the process of reaffiliation and formation of coffee fruits that are considered priority sinks and require large amounts of carbohydrates [4].

In summary, our results highlight the importance of seasonal climatic conditions in the growth and development of the conilon coffee plant, as well as in the accumulation of starch reserves in the leaves.

## 5. Conclusions

Genotype 143 has a shorter growth cycle and potentially a lower leaf production compared to the others. This may require adjustments in agronomic management practices, such as fertilization, particularly during critical periods like between the MAP (maximum accumulation of photosynthetic) and IP (initial phase), when nutritional demand is higher. The genotype P2, with a later onset of defoliation and a larger leaf area, indicates greater photosynthetic capacity and biomass accumulation, suggesting a superior productive potential.

Regardless of the harvest timing, all genotypes exhibited high starch levels in the summer and elevated total soluble sugars in the spring, meeting the developmental demands of fruits and leaves. Overall, the results suggest that the P2 genotype of *C. canephora* possesses favorable characteristics for greater leaf biomass production and growth potential compared to other genotypes, making it particularly promising for optimization in cultivation practices.

The different morphological and biochemical responses of the leaves among *C.canephora* genotypes in response to seasonal variations provide important insights for crop management, especially considering scenarios of climate change, particularly in full-sun cultivation areas. Furthermore, to the best of our knowledge, this study is pioneering in the application of the logistic model, taking critical points into account, to describe the leaf emission of coffee genotypes. No previous research has assessed the recovery of carbohydrate metabolism post-harvest, underscoring the relevance and innovation of our findings.

## Figures and Tables

**Table 1:** Characterization of the evaluation times of the leaves of early genotypes 104 and A1, intermediate P2, and late 143 in four seasons of the year.

Genotypes	Harvest	Reviews	Period Between Harvest and Last Evaluation
104 and A1	9 June	2 August (P55)	103 days
P2	29 June	23 August (I55)	83 days
143	27 July	20 September (T55)	55 days

**Table 2:** Critical points of the Logistics model. *a*, *b*, and *c* are the estimated parameters.

MAP	IP	MDP	ADP
$\frac{\cdot b-1.319}{c}$	*b*	$\frac{c\cdot b+1.319}{c}$	$\frac{c\cdot b+2.2924}{c}$
$\frac{a}{3+\sqrt{3}}$	$\frac{a}{2}$	$a\cdot \frac{3+\sqrt{3}}{6}$	$a\cdot \frac{a}{1.1010}$

**Table 3:** Average data of the meteorological parameters of maximum temperature (Max. T., °C), minimum temperature (Min. T., °C), average temperature (Avg. T., °C), average relative humidity (Humidity, %), accumulated precipitation (Precipitation, mm), solar radiation (Radiation, KJ/m^2^), potential evapotranspiration (ETp, mm $d^{-1}$) and photoperiod (Phot.) from August 2021 to August 2022.

Months	A	S	O	N	D	J	F	M	A	M	J	J	A	Annual
Max. T.	29.85	31.40	29.54	29.20	30.04	33.53	32.48	33.50	33.30	29.69	28.63	28.27	28.91	30.64 ^a^
Min. T.	17.85	20.50	21.44	19.95	21.11	21.96	22.27	21.26	19.97	17.60	15.28	16.25	16.89	19.41 ^a^
Avg. T.	25.66	27.13	25.46	23.79	24.67	26.65	25.93	26.10	25.27	22.31	20.50	20.87	21.02	24.26 ^a^
Humidity	68.00	65.10	73.48	77.12	77.10	70.73	75.57	72.11	69.68	71.16	71.42	73.31	68.85	71.82 ^a^
Precipitation	2.2	2.2	97.6	162.8	197.6	97.6	284.2	31.8	12	44.6	3.4	24.6	12.2	972.80 ^b^
Radiation	1266.07	1306.39	1130.56	1178.23	1138.92	1553.81	1352.86	1550.15	1350.12	1058.89	1085.43	1003.25	1183.27	1242.92 ^a^
ETp	125.63	143.04	135.64	143.92	153.43	182.80	146.95	163.98	139.89	108.57	95.27	98.54	112.94	1750.59 ^b^
Phot.	11.26	11.74	12.30	12.79	13.14	12.98	12.41	12.00	11.36	11.09	10.56	10.88	11.23	153.74 ^b^

^a^ Monthly average, ^b^ sum of monthly average.

**Table 4:** *p*-values of the Shapiro–Wilk (SW), Breusch–Pagan (BP), and Durbin–Watson (DW) tests and estimates of the fit of the data to the nonlinear Logistic model on the growth of the vegetative characteristics of the early maturation genotypes 104 and A1, intermediate P2, and late maturation 143 in different seasons of the year.

Genotype	SW	BP	DW	$R^{2}$	$R_{aj}^{2}$	AIC
104	0.4988	0.1287	0.104	0.97	0.96	32.4163
A1	0.7262	0.1685	0.108	0.98	0.98	31.5385
P2	0.4427	0.5668	0.050	0.99	0.99	25.5608
143	0.7686	0.0581	0.206	0.98	0.98	22.5035

**Table 5:** Growth variables in different seasons. Maximum width of the leaf blade (LW; cm), leaf central vein length (LL; cm), leaf area (LA; cm^2^), daily leaf area expansion rate (DLAFE; cm^2^ d^−1^), leaf dry mass (LDM; g), specific leaf area (SLA; cm^2^ g^−1^), leaves of plagiotropic branches (NL), early maturation genotypes 104 and A1, intermediate P2 and late 143.

Genotypes	Winter	Spring	Summer	Autumn	Averages
			Maximum width of the leaf blade (LW)		
104	1.34 Ab	1.44 Cb	2.29 Ba	1.64 Bb	1.68
A1	1.25 Ab	1.63 Cb	2.46 Ba	2.11 Aa	1.86
P2	1.29 Ac	2.35 Bb	3.11 Aa	2.41 Ab	2.29
143	1.23 Ac	3.03 Aa	2.12 Bb	1.52 Bc	1.98
Averages	1.28	2.11	2.50	1.92	
			Leaf central vein length (LL)		
104	2.70	3.16	6.65	4.85	4.34 a
A1	3.04	4.10	6.66	6.46	5.06 a
P2	2.73	5.58	7.14	5.63	5.27 a
143	3.02	6.11	6.35	4.37	4.96 a
Averages	2.87 C	4.74 B	6.7 A	5.33 B	
			Leaf area (LA)		
104	4.97	7.72	20.68	11.17	11.13 b
A1	5.12	11.18	22.78	18.40	14.37 b
P2	4.84	25.58	29.85	18.66	19.73 a
143	5.00	17.77	18.30	9.13	12.55 b
Averages	4.98 C	15.56 B	22.9 A	14.34 B	
			Daily leaf area expansion rate (DLAFE)		
104	0.06	0.75	2.23	0.86	0.97 b
A1	0.03	0.80	2.42	1.32	1.14 b
P2	0.15	2.26	3.08	1.33	1.71 a
143	0.06	1.48	1.95	0.58	1.02 b
Averages	0.07 C	1.32 B	2.42 A	1.02 B	
			Leaf dry mass (LDM)		
104	0.09	0.17	0.42	0.26	0.23 b
A1	0.06	0.24	0.37	0.33	0.25 b
P2	0.10	0.66	0.65	0.47	0.47 a
143	0.07	0.32	0.30	0.19	0.22 b
Averages	0.08 B	0.35 A	0.44 A	0.31 A	
			Specific leaf area (SLA)		
104	53.02	39.52	51.06	43.12	46.68 b
A1	91.72	46.60	62.18	55.59	64.02 a
P2	48.44	40.02	45.67	39.77	43.47 b
143	72.68	55.35	60.47	48.84	59.34 a
Averages	66.47 A	45.37 C	54.85 B	46.83 C	
			Leaves of plagiotropic branches (NL)		
104	0.25	6.79	4.33	0.00	2.84 a
A1	1.01	8.72	4.54	0.00	3.57 a
P2	2.64	5.38	4.74	0.08	3.21 a
143	1.92	5.75	0.95	0.00	2.16 a
Averages	1.45 C	6.66 A	3.64 B	0.02 D	

Means followed by the same lowercase letter in the row or uppercase letter in the column do not differ significantly from each other according to the Scott-Knott grouping test p≤0.05.

**Table 6:** Quantification of reducing sugars (RS, µmol/g, DM), total soluble sugars (TSS, µmol/g, DM) and starch (µmol/g, DM) of early maturation genotypes 104 and A1, intermediate P2 and late maturation genotypes 143 in four seasons of the year.

Genotypes	Winter	Spring	Summer	Autumn	Harvest	Averages
			RS			
104	634.31 Ba	385.6 Bc	372.31 Ac	480.08 Ab	318.86 Ac	547.79
A1	762.33 Aa	500 Ab	430.36 Ab	467.58 Ab	466.14 Ab	656.60
P2	651.11 Ba	400.69 Bb	439.6 Ab	424.15 Ab	561.03 Aa	619.15
143	565.55 Ba	354.71 Bb	430.37 Ab	414.49 Ab	557.24 Ba	580.59
Averages	653.33	410.25	418.16	446.58	475.82	
			SST			
104	2534	3443	3013	2686	1416	2618.25 a
A1	2782	3979	1828	2859	1594	2608.54 a
P2	2129	3079	2642	3248	2585	2736.67 a
143	2617	3086	2256	3129	2797	2777.05 a
Averages	2515.71 B	3396.74 A	2434.85 B	2980.28 A	2098.06 B	
			Starch			
104	2843	2757	3205	2954	2927	2936.86 a
A1	2775	3057	3116	2946	3083	2995.41 a
P2	2804	2866	3294	3094	3016	3014.71 a
143	2760	2677	2945	2973	2850	2840.81 a
Averages	2795.49 C	2839.24 C	3139.69 A	2991.51 B	2968.81 B	

Means followed by the same lowercase letter in the row or uppercase letter in the column do not differ significantly from each other according to the Scott-Knott grouping test p≤0.05.

**Table 7:** Quantification of reducing sugars (RS, µmol/g, DM), total soluble sugars (TSS, µmol/g, DM) and starch (µmol/g, DM) of early maturation genotypes 104 and A1, intermediate P2, and late 143 of leaves harvested 55 days after harvest (Aug 2 (P55), Aug 23 (I55), and Sep 20 (T55).

Genotypes	P55	I55	T55	Averages
		RS		
104	369.05 Ab	492.97 Aa	437.64 Ba	324.92
A1	480.56 Ab	528.37 Ab	622.69 Aa	407.91
P2	515.79 Aa	482.75 Aa	445.44 Ba	361.00
143	471.44 Aa	438.03 Aa	540.3 Aa	362.44
Averages	366.95	362.29	402.11	
		SST		
104	2336	2443	3340	2706.49 a
A1	2032	2589	3302	2641.02 a
P2	2164	2630	2679	2490.98 b
143	1974	2965	3104	2681.03 a
Averages	2126.47 C	2656.63 B	3106.53 A	
		Starch		
104	3136	2945	4422	3500.99 a
A1	3037	2975	4200	3404.11 a
P2	3255	3007	4261	3507.5 a
143	3046	3051	4311	34369.09 a
Averages	3118.35 B	2994.48 B	4298.44 A	

Means followed by the same lowercase letter in the row or uppercase letter in the column do not differ significantly from each other according to the Scott-Knott grouping test p≤0.05.

**Table 8:** Pearson’s correlation coefficients between leaf variables and climatic factors in four seasons of the year of early maturation genotypes 104 and A1, intermediate P2 and late 143 in four seasons of the year.

Variable	Etp	Radiation	Phot.	Max. T.	Min. T.	Avg. T.	Humidity	Starch	TSS	RS	NL	LL	LW	LA	LDM	SLA	DLAFE
Precip.	0.91 *	0.76 *	0.78 *	0.44	0.98 *	0.34	0.87 *	0.44	0.07	−0.67 *	0.69 *	0.66 *	0.66 *	0.71 *	0.6 *	−0.25	0.82 *
Etp		0.96 *	0.94 *	0.56 *	0.82 *	−0.02	0.91 *	0.61 *	0.12	−0.83 *	0.46	0.83 *	0.73 *	0.8 *	0.71 *	−0.40	0.88 *
Radiation			0.9 *	0.69 *	0.66 *	−0.17	0.78 *	0.71 *	0.03	−0.80 *	0.21	0.88 *	0.7 *	0.8 *	0.7 *	−0.39	0.85 *
Photo.				0.32	0.64 *	−0.32	0.95 *	0.49	0.34	−0.90	0.45	0.76 *	0.67 *	0.73 *	0.69 *	−0.54 *	0.76 *
Max. T.					0.47	0.28	0.15	0.75 *	−0.53 *	−0.26	−0.21	0.66 *	0.44	0.55 *	0.39	0.08	0.64 *
Min. T.						0.52 *	0.76 *	0.41	−0.04	−0.53 *	0.68 *	0.58 *	0.6 *	0.64 *	0.52 *	−1.13	0.77 *
Avg. T.							−0.13	0.00	−0.46	0.34	0.31	−0.11	0.00	0.00	−0.11	0.45	0.12
Humidity								0.34	0.40	−0.84 *	0.67 *	0.64 *	0.64 *	0.67 *	0.64 *	−0.50 *	0.72 *
Starch									−0.01	−0.35	0.07	0.56 *	0.27	0.33	0.13	−0.02	0.67 *
TSS										−0.29	0.42	−0.15	−0.13	−0.15	−0.06	−0.41	−0.12
RS											−1.35 *	−0.68 *	−0.65 *	−0.69 *	−0.70 *	0.74 *	−0.68 *
NL												0.06	0.13	0.14	0.09	−0.14	0.33
LL													0.89	0.93 *	0.80 *	−0.22	0.85 *
LW														0.92 *	0.84 *	−0.26	0.74 *
LA															0.95 *	−1.32	0.75 *
LDM																−0.47	0.57 *
SLA																	−0.12

* p≤0.05. Accumulated precipitation (Precip.); potential evapotranspiration (ETp); solar radiation (Radiation); photoperiod (Phot.); maximum temperature (Max. T.); minimum temperature (Min. T.); average temperature (Avg. T.); average relative humidity (Humidity); starch; total soluble sugars (TSS); reducing sugars (RS); plagiotropic branch leaves (NL); Leaf midrib length (LL); maximum leaf blade width (LW); leaf area (LA); leaf dry mass (LDM); specific leaf area (SLA); daily leaf area expansion rate (DLAFE).

## Data Availability

The original contributions presented in the study are included in the article, further inquiries can be directed to the corresponding authors.
